# Editors’ Book Table

**Published:** 1872-07

**Authors:** 


### Editors' Book Table.

[NOTE. — All works reviewed in the columns of the CHICAGO MEDICAL JOURNAL may be found in the extensive stock of W. B. KEEN, COOKE & CO., whose catalogue of Medical Books will be sent to any address upon request.]

BOOKS RECEIVED.

*Lectures on the Principles and Practice of Physic.* Delivered at King's College, London, by Sir THOMAS WATSON, Bart., M.D., F.R.S., etc., etc. A new American, from the fifth revised and enlarged English edition. Edited, with additions, and several hundred illustrations, by HENRY HARTSHORNE, M.D., Professor of Hygiene in the University of Pennsylvania. In two large and handsome 8vo. vols. Cloth, $9.00; leather, $11.00.

With the assistance of Professor George Johnson, his successor in the chair of Practice of Medicine in King's College, the author has thoroughly revised this work, and has sought to bring it on a level with the most advanced condition of the subject. As he himself remarks: "Considering the rapid advance of medical science during the last fourteen years, the present edition would be worthless, if it did not differ much from the last" — but in the extensive alterations and additions that have been introduced, the effort of the author has been to retain the lucid and colloquial style of the lecture-room, which has made the work so deservedly popular with all classes of the profession. Notwithstanding these changes, there are some subjects on which the American reader might reasonably expect more detailed information than has been thought requisite in England, and these deficiencies the editor has endeavored to supply.

The large size to which the work has grown seems to render it necessary to print it in two volumes, in place of one, as in the last American edition. It is therefore presented in that shape, handsomely printed, at a very reasonable price, and it is hoped that it will fully maintain the position everywhere hitherto accorded to it, of the standard and classical representative of English practical medicine. (*Publisher's Notice*).

We are exceedingly gratified at the reception of this new edition of Watson, pre-eminently the prince of English authors, on "Practice." We who read the first edition as it came to us tardily and in fragments through the "Medical News and Library," shall never forget the great pleasure and profit we derived from its graphic delineations of disease, its vigorous style and splendid English, reminding the reader of the better days of the Republic of Letters. There was scarcely any nonsense in the whole book, and what little there was did not come from Dr. Watson himself. Maturity of years, extensive observation, profound research, and yet continuous enthusiasm, have combined to give us in this latest edition a model of professional excellence in teaching, with rare beauty in the mode of communication. But this *classic* needs no eulogium of ours.

The selection of Prof. Hartshorne as the American editor, is to us peculiarly gratifying, and must ensure even larger popularity and more general sale to American readers. Every guarantee is thus afforded that in every part the book will be found up to the times. Will it do to repeat the remark we have seen somewhere: "No library can be considered complete without it"? Although the phrase may not savor of originality, it is, nevertheless, most emphatically true.

*The Correct Principles of Treatment for Angular Curvature of the Spine.* By BENJAMIN LEE, A.M., M.D. Philadelphia: J. B. Lippincott & Co. 1872.

Several years since, Dr. Lee published a little volume entitled "Contributions to the Diagnosis, Pathology and Treatment of Angular Curvature of the Spine," now out of print. The present essay is to a certain extent a new edition, omitting that which has now passed out of the pale of controversy, or was indirectly personal. It is a very useful monograph on an important subject.

PAMPHLETS.

*Spectrum Analysis Discoveries:* Showing its Application in Microscopical research, and to discoveries of the Physical Constitution and Movements of the Heavenly Bodies. From the Works of Schellen, Young, Roscoe, Lockyer, Huggins and others. Boston: Lee & Shepard. New York: Lee, Shepard & Dillingham. Price, 25 cts.

This is No. 4 of the series edited by Dana Estes, entitled "Half-Hour Recreations in Popular Science." Twelve Nos. are issued annually, at 25 cts. each, or $2.50 a year. The present number is beautifully illustrated.

*The Nes Silicon Steel.* E. GULICK, Manager and Attorney, Rome, N. Y.

*The Better Way; Or, Considerations upon the Natural System of Providing for the Treatment of the Insane.* By ANDREW McFARLAND, M.D., LL.D. Pp. 40.

*An American Italy for Invalids:* — A Dissertation showing the Advantages, Incidents, etc., of a Journey on the Plains, in the Rocky Mountains and Mexico, for the Cure of all Chronic Diseases. By R. E. FULLERTON, M.D. June, 1872. Vol. 1, No. 1, pp. 64. Mailed to any address by H. D. Chapin & Co., Booksellers, 50 East Harrison Street, Chicago. Price, 25 cts.

